# Exploring Pathophysiological Processes Shared Between Type 2 Diabetes Mellitus and Osteoarthritis

**DOI:** 10.3390/ijms27209168

**Published:** 2026-10-16

**Authors:** Yasir Majeed, Gundula Gesine Schulze-Tanzil, Maria Kokozidou

**Affiliations:** Institute of Anatomy and Cell Biology, Paracelsus Medical Private University Nuremberg and Salzburg, 90419 Nuremberg, Germany; ymajeed49@gmail.com (Y.M.); gundula.schulze@pmu.ac.at (G.G.S.-T.)

**Keywords:** osteoarthritis, diabetes mellitus, shared links, hyperglycemia, cartilage

## Abstract

Type 2 diabetes mellitus (T2DM) and osteoarthritis (OA) are highly prevalent chronic disorders with increasing global burdens that frequently coexist and share common risk factors, including obesity, aging and chronic low-grade inflammation. This narrative review aims to summarize current evidence regarding the epidemiological, clinical and molecular links between T2DM and OA and to highlight potential therapeutic implications. Growing evidence suggests that T2DM contributes to OA development and progression through multiple interconnected mechanisms. These include advanced glycation end-product (AGE) accumulation, oxidative stress, chronic inflammation, extracellular matrix degradation, impaired autophagy, ferroptosis, mesenchymal stem cell dysfunction and microvascular impairment within joint tissues. In addition, shared genetic and molecular pathways, including AGE–RAGE, IL-17, PI3K/Akt/mTOR and LGR6/Wnt/β-catenin/NRF2 signaling, further support a biological connection between the two diseases. Clinically, T2DM is associated with increased OA severity, enhanced pain sensitization, accelerated disease progression and higher rates of joint arthroplasty. Emerging evidence also suggests that antidiabetic agents, particularly metformin and glucagon-like peptide 1 receptor agonists, may exert disease-modifying effects through anti-inflammatory, metabolic and chondroprotective mechanisms. Overall, T2DM and OA should be regarded as interconnected metabolic-inflammatory disorders. Improved understanding of their shared mechanisms may facilitate earlier intervention, therapeutic repurposing and integrated multidisciplinary management strategies to reduce disease burden and improve patient outcomes.

## 1. Introduction

Osteoarthritis (OA) is the most prevalent form of arthritis and a leading cause of pain, disability and reduced quality of life worldwide. It affects over 500 million people globally, predominantly those over 55 years of age, with a higher prevalence among women [1,2,3,4]. The knee is the most frequently affected joint [5]. Once considered a degenerative “wear-and-tear” disorder, OA is now recognized as a complex, whole-joint disease resulting from interactions between mechanical, biological and systemic factors [6]. Pathological changes extend beyond articular cartilage degradation and include synovitis, subchondral bone remodeling and sclerosis, osteophyte formation, meniscus and ligament degeneration and alterations in periarticular muscle structure [7,8,9].

Type 2 diabetes mellitus (T2DM), the most common form of diabetes, is a chronic metabolic disorder characterized by insulin resistance, progressive β-cell dysfunction and persistent hyperglycemia affecting more than 10% of the adult population worldwide. T2DM is associated not only with classical microvascular and macrovascular complications but also with significant musculoskeletal manifestations [10]. Chronic low-grade inflammation, oxidative stress, metabolic dysregulation and microvascular dysfunction are recognized hallmarks of T2DM that may influence the integrity and function of connective tissues. There is a constant rise in the prevalence of both diseases [11,12]. Increasing evidence suggests that OA and T2DM are interconnected diseases rather than merely age-related conditions, with shared risk factors such as obesity, physical inactivity and chronic inflammation contributing to the development of both disorders, while epidemiological studies demonstrate that individuals with T2DM have an increased risk of developing OA independent of age and obesity [13]. While obesity and physical inactivity represent important, shared risk factors for both conditions, accumulating evidence suggests that metabolic abnormalities inherent to T2DM may directly influence the initiation and progression of OA [13,14]. Consequently, the concept of a distinct “metabolic OA phenotype” has emerged, proposing that metabolic disturbances can actively contribute to joint degeneration independent of excessive mechanical loading [15,16].

Beyond epidemiological associations, OA and T2DM share several molecular and cellular mechanisms, including chronic inflammation, oxidative stress, extracellular matrix (ECM) remodeling, mitochondrial dysfunction, altered cellular metabolism and dysregulated signaling pathways [17,18,19,20,21,22]. Recent transcriptomic and bioinformatic studies have further identified differentially expressed genes (DEGs) and common effector pathways that may contribute to the pathogenesis of both diseases [17,18,23].

These findings suggest that the coexistence of OA and T2DM may not merely reflect the presence of common risk factors, but rather a biologically relevant interaction in which metabolic dysfunction promotes joint degeneration, while chronic musculoskeletal impairment further worsens systemic metabolic health.

The aim of this review is to summarize current evidence regarding the interplay between T2DM and OA, with particular emphasis on their epidemiological associations, shared risk factors and underlying molecular mechanisms. We specifically discuss common inflammatory and immune pathways, metabolic alterations, ECM remodeling and emerging gene networks implicated in both diseases [17,18,19,20,21,22,23]. By elucidating these interconnected mechanisms, this review aims to provide a framework for future research and support the development of integrated therapeutic strategies targeting both metabolic and musculoskeletal health, ultimately improving patients’ outcomes and informing public health approaches.

## 2. Shared Risk Factors and Epidemiological Links

OA is the most common form of arthritis and a major contributor to global disability [24]. The perception of OA has shifted from a predominantly degenerative cartilage disorder to a complex whole-joint disease involving structural and functional alterations across multiple joint tissues [6,7,8]. This shift reflects recognition that OA pathogenesis integrates biomechanical factors with metabolic, inflammatory and systemic processes, many of which overlap with the biological disturbances’ characteristic of T2DM. Both OA and T2DM are widely known as metabolism-affected diseases that share major common risk factors such as obesity and aging [25], while biological sex influences disease susceptibility and progression [26].

### 2.1. Obesity

Obesity remains one of the strongest shared risk factors linking T2DM and OA. In OA, excess body weight increases mechanical loading of weight-bearing joints, particularly the knees and hips, thereby accelerating cartilage degeneration and altering subchondral bone remodeling [27,28,29,30]. Consistent with this, a large cohort study involving 1,764,061 subjects revealed that obese individuals with grade 2 obesity were 4.7-fold more frequently diagnosed with knee OA (KOA) compared to healthy subjects independent of background pathology [31]. Furthermore, the clinical importance of obesity in OA in a randomized controlled trial by Messier and colleagues revealed that dietary adjustments and exercise led to a statistically significant reduction in knee pain over an 18-month period in individuals with KOA who were overweight or obese, with body mass index (BMI) > 27, compared to the control group [32].

Obesity is one of the most important and well-established risk factors for T2DM. In a large prospective cohort study of 84,941 women followed for 16 years, overweight and obesity (BMI > 25) emerged as the strongest predictors of incident T2DM [33]. Additional longitudinal studies demonstrated that both the duration and severity of obesity contribute to T2DM risk, emphasizing the cumulative metabolic consequences of prolonged excess adiposity [34].

Beyond its mechanical effects on joints and its contribution to insulin resistance, obesity provides a biological link between OA and T2DM, since adipose tissue is now recognized as an active endocrine organ that produces proinflammatory adipokines, cytokines, chemokines and free fatty-acids promoting chronic low-grade systemic inflammation, a hallmark of both T2DM and OA [35,36,37].

Consequently, obesity may contribute to OA not only through increased mechanical load but also through systemic metabolic and inflammatory mechanisms.

This concept has led to the emergence of a metabolic OA phenotype, in which obesity, metabolic syndrome and low-grade inflammation actively contribute to joint degeneration beyond the effects of mechanical stress alone [16]. Supporting this concept, Schett et al. demonstrated in a population-based longitudinal cohort that T2DM was an independent predictor of severe OA after adjustment for age and BMI, suggesting that metabolic dysfunction contributes directly to disease progression [38]. These observations indicate that obesity serves not only as a shared epidemiological risk factor but also as a central biological mediator linking T2DM and OA.

The clinical consequences of this interaction are increasingly evident in a recent cross-sectional study that reported that individuals with both T2DM and symptomatic KOA experienced significantly lower health-related quality of life (HRQoL) than those without OA, largely due to increased pain, sleep disturbances, depressive symptoms, fatigue and walking limitations [39].

In summary, obesity represents a major shared risk factor for both T2DM and OA through a combination of mechanical, metabolic and inflammatory mechanisms. While obesity contributes substantially to the epidemiological overlap between these diseases, accumulating evidence suggests that obesity-associated metabolic disturbances may contribute to OA development and progression through several biologically plausible pathways.

### 2.2. Fatty Acids and Cartilage Degeneration

The contribution of adipose tissue extends beyond systemic inflammation and contributes to the production of free fatty acids (FFAs). FFAs can be classified according to their degree of saturation as saturated fatty acids (SFAs), monounsaturated fatty acids (MUFAs) and polyunsaturated fatty acids (PUFAs). The relationship between a diet rich in SFAs and the radiographic progression of knee OA has also been studied in a clinical setting [40]. The results suggested that higher dietary SFA levels could be associated with the progressively decreased knee joint space, related to the progressive cartilage loss observed over four-year follow-up periods [40].

Another study involving 5328 participants also resulted in similar findings, in which plasma fatty acid levels, including PUFA levels, were positively associated with structural knee OA, assessed by magnetic resonance imaging (MRI) [41]. SFAs and PUFAs enhance cartilage damage by increasing cell apoptosis and expression of genes related to cartilage degradation [41,42].

Together, those findings suggest that disturbances in lipid metabolism may contribute to cartilage degeneration independently of mechanical loading.

### 2.3. Adipose Tissue, Fat Distribution and Metabolic Inflammation

Low-grade inflammation represents a central biological link between OA and T2DM, since both diseases are characterized by elevated levels of proinflammatory mediators, particularly interleukin (IL)-6 and tumor necrosis factor (TNF)-α, which contribute to metabolic dysfunction and joint degeneration in both animal and human models [43,44,45,46]. Moreover, an animal study showed that the peroxisome proliferator-activated receptor gamma (PPARγ) is involved in inflammation associated with both OA and T2DM [47].

The infrapatellar fat pad (IFP) is frequently underrepresented in OA research, but it plays a significant role as an active local player in KOA pathology. The IFP becomes inflamed and fibrotic in OA [48,49]. Moreover, the IFP also functions as a local source of inflammatory cytokines such as IL-6, adipsin, adiponectin and visfatin, which promote joint inflammation and facilitate both peripheral and central sensitization, exacerbating pain in KOA [49,50]. As observed in a mouse model, hyperglycemic conditions promote fibrogenic differentiation of the IFP and IL-6 production [51].

The importance of fat distribution patterns, particularly visceral fat, in the development of OA, especially in individuals with T2DM or prediabetes, has also been recently highlighted [52]. The Body Roundness Index (BRI) and the Visceral Fat Metabolism Score (METS-VF) are emerging as significant indicators of metabolic health and are closely associated with OA risk. The study by Ye et al., using National Center for Health Statistics of the Centers for Disease Control and Prevention (NHANES) data from 1999 to 2020, looked at people with diabetes mellitus (DM) or prediabetes (PD) [52]. They reported that those who had OA showed significantly higher BRI and METS-VF values compared to people without OA (BRI: 6.61 ± 0.17 vs. 5.70 ± 0.07, *p* < 0.0001; METS-VF: 7.26 ± 0.04 vs. 6.98 ± 0.02, *p* < 0.0001). Each unit increase in BRI was associated with a 15% higher risk of developing OA, and each unit increase in METS-VF was linked to a 133% increase in OA risk (*p* < 0.0001) [52]. The study also demonstrated a non-linear relationship between METS-VF and OA risk, while BRI exhibited a linear relationship with OA risk. However, combining BRI and METS-VF did not show significant added predictive value over either measure alone, suggesting that while both are strong predictors, they independently provide meaningful insights into OA risk [52].

### 2.4. Insulin Resistance, Hyperinsulinemia and Cartilage Homeostasis

Insulin therapy is a common treatment strategy in T2DM, and it has been shown to be associated with articular cartilage degeneration [53,54]. Insulin enhances the production of inflammatory cytokines such as TNF-α and IL-6, and consequently, the chronic low-grade inflammation associated with insulin resistance exacerbates the degradation of joint cartilage [55,56]. Insulin signaling plays an important role in cartilage metabolism and chondrocyte survival, while chronic hyperinsulinemia may adversely affect cartilage homeostasis, a fact that has been verified in both in vivo and in vitro studies on primary chondrocytes, where elevated insulin reduced cell viability, decreased metabolic activity and lowered proteoglycan synthesis, the latter suggesting that ECM maintenance was impaired [57,58,59]. These mechanisms could impair cartilage maintenance by inhibiting chondrocyte maturation [60]. Moreover, hyperinsulinemia has also been found to be associated with a lower triiodothyronine/thyroxin (T3/T4) ratio, suggesting impaired conversion of T4 to T3 [61]. Given the fact that T3 is essential for chondrocyte differentiation and maturation, such alterations in thyroid hormones may impair cartilage homeostasis and potentially contribute to OA pathogenesis and predisposition [62,63]. Recent Mendelian randomization (MR) evidence strengthens the assumption of a causal role of insulin in OA risk [64]. In an analysis involving up to 39,427 OA cases and 378,169 controls, genetically proxied insulin therapy was associated with an increased risk of overall OA and hip OA (HOA), with associations persisting after adjustment for confounders including age, sex, BMI, alcohol consumption and smoking status. This finding was independent of mediators that predispose a person to OA (such as obesity), suggesting a direct genetic link rather than solely indirect effects through metabolic factors [64].

### 2.5. Aging and Genetic Links

Aging is another major common risk factor for both T2DM and OA, and the prevalence of both diseases increases substantially with age [65,66,67]. Epidemiologically, the coexistence of these disorders is striking; according to the Centers of Disease Control and Prevention (CDC), approximately 47.3% of individuals with T2DM have a physician-diagnosed OA, with a higher incidence in the individuals over 45 years of age [68].

Genetic studies provide additional support for a biological relationship between the two diseases [23]. T2DM and OA share 18 colocalized genomic risk loci, with the most prevalent being the obesity-related fat mass and obesity-associated gene (FTO) locus, (95%). Increased expression of the Iroquois homeobox 3 (IRX3) gene, which regulates adipogenesis, was observed in both pancreatic islets and the synovium. Two high-confidence effector genes, cysteine-rich wall integrity and stress component domain-containing 2 (WSCD2) gene and transmembrane protein 119 (TMEM119), were identified at the same locus and showed colocalization for both KOA and T2DM [23]. There is a stronger genetic correlation for T2DM and KOA than for HOA and T2DM [23]. Other groups have reported that differentially expressed genes such as matrix metalloproteinase (MMP)-9 and angiopoietin-like (ANGPTL) 4 were related to both T2DM and OA [17]. A larger study identified a total of 12 genes, namely, ephrin type-A receptor (EPHA) 3, RAS interaction protein (RASIP) 1, proenkephalin (PENK), leucine-rich repeat-containing (LRRC) 17, CCAAT/enhancer-binding protein beta (CEBPB), EGF-like fibulin ECM protein (EFEMP) 2, ubiquitin-associated protein (UBAP) 1, protein phosphatase 1 regulatory subunit (PPP1R) 15A, solid pseudopapillary epithelial neoplasm (SPEN), v-maf avian musculoaponeurotic fibrosarcoma oncogene homolog (MAF) F, growth arrest and DNA damage-inducible beta (GADD45B) and Krüppel-like factor (KLF) 4 shared by both diseases and further demonstrated that these genes are involved in important signaling pathways like IL-17, protein (p)53, nuclear factor kappa-light-chain-enhancer of activated B cells (NF-κB) and mitogen-activated protein kinase (MAPK) [19] (Figure 1).

These findings suggest that common genetic and molecular mechanisms may particularly explain the frequent coexistence of OA and T2DM.

In summary, the relationship between T2DM and OA can be viewed across several levels of evidence. First, the two diseases share major epidemiological risk factors, including obesity, aging and chronic inflammation. Second, observational studies consistently demonstrate an association between T2DM and increased OA prevalence and severity. Third, experimental and molecular studies provide biological plausibility by identifying common, inflammatory, metabolic and degenerative pathways. Finally, emerging genetic and MR studies suggest that some diabetes-related mechanisms may contribute directly to OA susceptibility. Together, these findings support a close biological relationship between T2DM and OA while highlighting the need for further studies to clarify the extent to which this association reflects shared risk factors versus true causal mechanisms.

## 3. Molecular and Cellular Mechanisms Linking T2DM and OA

### 3.1. Local and Systemic Inflammation

OA is characterized by both local joint inflammation and systemic inflammatory activation [6,69]. Articular cartilage injury and ECM fragmentation stimulate chondrocytes and synoviocytes to release proinflammatory mediators, including IL-1β, TNF-α, reactive oxygen species (ROS), nitric oxide (NO) and prostaglandins (PGs), which promote proteolytic enzyme production, including MMPs, thereby contributing to ECM degradation and progressive cartilage destruction [70,71,72,73].

T2DM is also associated with low-grade systemic inflammation, and experimental studies have shown that hyperglycemia can increase the expression of IL-1β, IL-6, and TNF-α, thereby activating NF-κB signaling pathways, which enhance catabolic activity in articular chondrocytes and ECM degradation–thus potentially accelerating OA progression [71,72,74,75].

Hyperglycemia may further amplify inflammatory signaling through increased oxidative stress and cytokine production within joint tissues [54,69]. In addition, obesity, a major shared risk factor for both T2DM and OA, contributes to systemic inflammation through the secretion of adipokines such as leptin by the adipose tissue. These adipokines can stimulate the production of ECM-degrading enzymes such as MMP-1, MMP-3 and MMP-13, while also promoting inflammatory signaling and chondrocyte apoptosis [76,77,78].

Collectively, these observations support the biological plausibility of the hypothesis that metabolic and inflammatory alterations associated with T2DM and obesity may contribute to joint degeneration, although the relative contribution of each pathway in diabetic OA requires further clarification (Figure 2).

### 3.2. Mitochondrial and Endoplasmic Reticulum (ER) and Oxidative Stress

Mitochondria are cellular organelles that regulate ATP production, ROS generation and apoptosis. Their dysfunction in OA causes oxidative stress, chondrocyte apoptosis and ECM degradation [79]. As mitochondrial dysfunction progressively develops and energy homeostasis gets out of balance, cartilage degeneration results [80]. At the same time, endoplasmic reticulum (ER) stress, which is characterized by the accumulation of misfolded or unfolded proteins [81], affects chondrocytes, synoviocytes and mesenchymal stem cells (MSCs) in the case of OA [82]. When ER stress persists, the unfolded protein response (UPR) is activated [81] and apoptosis and autophagy dysregulation follow in OA [83].

In the case of T2DM, ER stress is responsible for UPR activation, induced by the accumulation of large amounts of unfolded proteins, and leads to insulin resistance, pancreatic β-cell dysfunction and chronic inflammation [84,85]. ER stress contributes to cellular damage through multiple mechanisms. It increases ROS production, leading to mitochondrial dysfunction, while activation of the UPR sensors protein kinase RNA-like ER kinase (PERK), inositol-requiring enzyme 1 alpha (IRE1α) and the activating transcription factor 6 (ATF6) promotes oxidative stress and ultimately induces chondrocyte apoptosis [86].

In conclusion, ER and mitochondrial stress are well-established contributors to cartilage degeneration in OA and play important roles in the pathophysiology of T2DM. However, direct evidence demonstrating that these mechanisms specifically drive OA progression in patients with T2DM remains limited, and further studies using diabetic OA models and human tissues are required.

### 3.3. Glycation and ECM Alterations

Hyperglycemia can affect the cartilage ECM through post-translational modifications, such as glycation, glycoxidation, and nitrosylation [87]. These modifications disrupt the structural integrity of collagen–proteoglycan networks and may enhance enzymatic ECM degradation [87]. Among those changes, advanced glycation end products (AGEs) have attracted attention because they accumulate in both T2DM and aging and have been shown to impair chondrocyte function, leading to reduced ECM repair, increased catabolic activity and progressive cartilage degradation [88].

In both OA and T2DM, increased AGE accumulation has been associated with alterations in cartilage homeostasis. AGE-mediated collagen cross-linking modifies the biomechanical properties of the ECM, leading to increased tissue stiffness and reduced elasticity, changes that may contribute to cartilage degeneration and OA progression [88,89,90,91]. Importantly, these processes are not unique to T2DM but are also characteristic of aging, and the shared presence of oxidative stress, AGE accumulation, mitochondrial dysfunction and chronic low-grade inflammation further supports the biological overlap between T2DM-associated cartilage degeneration and age-related chondrosenescence [66,92].

Experimental evidence also supports an adverse effect of diabetes-associated metabolic disturbances on cartilage. In a rat streptozotocin-induced model of DM, reduced collagen synthesis and increased expression of proteoglycan-degradation enzymes were observed in articular cartilage [93,94]. While these findings suggest a potential contribution of hyperglycemia to cartilage degeneration, their direct translation to human diabetic OA requires further investigation.

Hyperglycemia may additionally disrupt cellular glucose transport mechanisms, and its accumulation further increases oxidative stress. Rosa and colleagues demonstrated that osteoarthritic chondrocytes obtained from older donors (≥66 years) and cultured under high-glucose conditions failed to downregulate glucose transporter 1 (GLUT1) expression or reduce glucose transport activity, in contrast to normal chondrocytes derived from younger donors (28–35 years) [58,95]. Moreover, it was also found that exposure of chondrocytes to a high-glucose environment favored production of ROS and promoted ECM catabolism, suggesting that hyperglycemia may contribute to OA progression [58,70,72,95].

Collectively, these observations support the biological plausibility that hyperglycemia and AGE accumulation may contribute to cartilage degeneration through alterations in ECM structure, oxidative stress and impaired chondrocyte function. However, much of the available evidence originates from experimental models or high-glucose culture systems and additional studies in human diabetic OA tissues are required to define the precise contribution of these mechanisms to disease progression (Figure 2).

### 3.4. Stem Cell Dysfunction and Impaired Chondrogenesis

Adequate glucose availability is required for proteoglycan synthesis and plays a critical role in ECM synthesis and maintenance of the chondrogenic phenotype [57,58,96]. Bone marrow MSCs are important reservoirs for the damage repair process in joints, as they have the capacity to undergo chondrogenic differentiation [97].

Experimental evidence suggests that diabetes-associated metabolic disturbances may affect this regenerative potential. In an in vitro study, Kume et al. showed that exposing human MSCs to AGEs interfered with their chondrogenic differentiation of human MSCs [98]. Similarly, high-glucose environments not only drive a marked rise in prostaglandin E2 (PGE2), MMP-13 and IL-6 production, but also suppress the synthesis of both PPARγ, which is involved in hyperglycemia-induced inflammation responses in human articular chondrocytes derived from the femoral head and cartilage-specific type II collagen (COL2) [47]. These findings suggest that hyperglycemic conditions may promote a more catabolic cellular phenotype.

PPARγ is an important regulator of cartilage homeostasis, as experimental studies have shown that its genetic deletion results in spontaneous and accelerated OA characterized by increased expression of inflammatory mediators, MMP-13, disintegrin and metalloprotease with thrombospondin motifs (ADAMTS)-5, chondrocyte apoptosis and enhanced cartilage degradation, indicating that physiological PPARγ activity normally suppresses inflammatory and catabolic pathways and limits the release of cartilage-degrading factors [99,100]. Consequently, suppression of PPARγ signaling under hyperglycemic conditions has been proposed as one potential mechanism linking metabolic dysfunction to impaired homeostasis.

Evidence from human studies remains limited. Cassidy et al. reported that patients with T2DM have fewer osteoprogenitor MSCs than age-matched controls. However, the remaining MSCs largely retained their proliferative, differentiation, migratory and angiogenic capacities. These findings suggest that T2DM-associated musculoskeletal complications may be related primarily to a decrease in osteoprogenitor cell numbers rather than a generalized impairment of MSC function [101].

In addition to bone marrow-derived MSCs, several other stem and progenitor cell populations may contribute to cartilage maintenance and repair. Xu et al. described cartilage stem/progenitor cells (CSPCs) that are localized in the superficial cartilage zone and possess MSC-like properties such as multipotency, clonogenicity and migration capacity [102]. Experimental studies in a diabetic rat model demonstrated that hyperglycemia changed the morphology of cartilage superficial layer cells and their interactions with the ECM, providing clear evidence of superficial zone cell stress [103]. However, the direct effects of T2DM-associated metabolic disturbances on CSPC biology remain poorly understood.

The synovium represents another important source of MSCs with high proliferative and chondrogenic potential compared with many alternative tissue sources [104,105]. Although these cells are increasingly investigated for cartilage repair applications, their specific responses to the diabetic metabolic environment have not yet been adequately characterized.

Likewise, the IFP contains a distinct MSC population with robust chondrogenic potential and stable multilineage differentiation capacity, suggesting a role in cartilage maintenance and joint homeostasis [106,107]. While studies involving adipose tissue-derived stem cells (ADSCs) indicate that hyperglycemia can impair osteogenic and chondrogenic differentiation and promote adipogenesis, it remains unclear whether IFP-derived MSCs exhibit similar responses given their unique anatomical location and biological characteristics [108,109].

Collectively, available evidence supports the biological plausibility that hyperglycemia, AGEs and other T2DM-associated disturbances may influence stem cell-mediated cartilage repair and chondrogenic differentiation. However, much of the current evidence from human diabetic OA tissues and disease-specific animal models remains limited, particularly for CSPCs, synovial MSCs and IFP-derived MSCs, highlighting an important area for future investigation.

### 3.5. Autophagy Suppression

Autophagy is a cellular homeostasis process responsible for maintaining metabolic balance by removing damaged intracellular products, which are sequestered in autophagosomes for degradation and utilization [53]. Autophagy is essential for chondrocyte survival and homeostasis. OA decreases autophagy, which enhances apoptosis and cartilage degeneration, contributing to a vicious circle [110]. In the case of T2DM, high glucose levels also decrease chondrocyte autophagy via inhibition of the adenosine monophosphate protein kinase (AMPK) pathway [53,111] and activation of mechanistic target of rapamycin (mTOR) signaling pathways [112].

Ribeiro et al. demonstrated that insulin suppresses autophagy in human chondrocytes, as evidenced by reduced microtubule-associated protein 1 light chain 3 (LC3 II) expression and activation of the protein kinase B (Akt)/mTOR signaling pathway, including increased phosphorylation of ribosomal protein S6 (rpS6), suggesting that insulin-mediated activation of mTOR negatively regulates chondrocyte autophagy and may contribute to cartilage degeneration [53,112,113]. Clinically, OA patients with T2DM exhibit lower LC3 II and higher phosphorylated (p)-rpS6 expression than either healthy controls or non-diabetic OA patients [53], consistent with reduced autophagic activity (Figure 2).

It should be noted that much of the evidence linking autophagy suppression to diabetic OA originates from high-glucose in vitro experiments or independent studies of OA and T2DM. Although these findings support a plausible mechanistic connection, direct confirmation in human diabetic OA tissues remains relatively limited.

### 3.6. Ferroptosis

Ferroptosis, which is a form of iron-dependent regulated cell death characterized by lipid peroxidation and oxidative stress, is now emerging as one of the mechanisms in OA pathogenesis, in which human OA chondrocytes exhibit ferroptotic changes such as intracellular iron accumulation, increased ROS production and elevated expression of cartilage-degrading enzymes, while pharmacological inhibition of ferroptosis attenuates those effects [114,115].

This is markedly distinct from conventional cell death models such as apoptosis and necrosis, as it neither has the morphological characteristics of necrosis (such as swelling of the cytoplasm and organelles and rupture of the cell membrane) nor does it follow the traditional cell apoptosis pattern (such as cell shrinkage, chromatin condensation, formation of apoptotic bodies and disintegration of the cytoskeleton) [116]. Ferroptosis is characterized by iron accumulation and lipid peroxidation (LPO), with morphological features such as mitochondrial shrinkage with increased mitochondrial membrane density, and the reduction or disappearance of mitochondrial cristae [116].

The role of leucine-rich repeat-containing G-protein-coupled receptor 6 (LGR6)-related pathway is also interesting, as it is emerging as a key modulator of ferroptosis in the DM-OA axis [117]. Chen et al. studied the role of LGR6 in ferroptosis using bioinformatic analysis of transcriptomic data from OA and diabetic OA (DM-OA) mouse models. They constructed functional enrichment and protein–protein interaction (PPI) networks. In vivo, diabetic OA was modeled in mice by high-fat diet/streptozotocin induction combined with destabilization surgery on the medial meniscus. In vitro, chondrocytes were exposed to high glucose levels to mimic diabetic conditions [117]. They identified a total of 16 genes associated with OA progression, and the genes encoding catabolic factors, such as MMPs (MMP-3 and MMP-13) and nitric oxide synthase-2 (NOS2), were found to be upregulated in DM-OA chondrocytes. Genes encoding bone morphogenetic protein 2 (BMP2), COL2A1, insulin-like growth factor (IGF)1 and IL-4 were found to be downregulated in DM-OA. Notably, LGR6 was significantly downregulated, suggesting a connection between LGR6 and DM-OA in vivo. They demonstrated that LGR6 expression is significantly downregulated in diabetic cartilage and that its loss exacerbates, while its overexpression ameliorates, OA progression by modulating chondrocyte ferroptosis. They also proposed that LGR6 modulates ferroptosis through the Wnt/β-catenin pathway [117]. LGR6 activation is an established potentiator of Wingless-related integration proteins (Wnt) signaling via R-spondin binding to stabilize β-catenin and promote its nuclear translocation. β-catenin-dependent transcription further activates antioxidant genes. Nuclear factor erythroid 2-related factor 2 (NRF2) is an important downstream mediator of this pathway and a master regulator of the antioxidant response. NRF2 stabilization leads to transcription of ferroptosis defense genes, including glutathione peroxidase 4 (GPX), suppresses proinflammatory and pro-oxidant mediators such as prostaglandin-endoperoxide synthase 2 (PTGS) and protects chondrocytes from ferroptosis [117,118]. Disruption of antioxidant pathways, including those involving GPX4 and cellular redox homeostasis, promotes ROS accumulation, lipid peroxidation and chondrocyte death, while emerging studies further indicate that LGR6-mediated signaling may exert a protective role through interactions with β-catenin and NRF2 pathways, although its contribution to DM-associated OA requires further investigation [99,119] (Figure 2).

Although ferroptosis has been increasingly recognized as a contributor to OA pathogenesis, evidence specifically linking ferroptosis to diabetic OA is still emerging. Current support is derived primarily from experimental diabetic OA models and bioinformatic analyses, and additional validation in human tissues is required.

### 3.7. Vascular Dysfunction as a Shared Pathway

Microvascular dysfunction, a hallmark of T2DM, is also implicated in OA pathophysiology [120,121]. Adequate subchondral bone perfusion is essential for nutrient exchange between bone and cartilage. T2DM-related vascular compromise impairs this process, limiting bone remodeling and disrupting bone–cartilage crosstalk [121,122]. These changes may contribute to cartilage degeneration and OA progression.

Recent evidence also indicates that T2DM is associated with profound microvascular dysfunction within the synovial tissue [123,124]. In patients with T2DM, the normally dense capillary network present in the synovial lining and sublining layers is markedly reduced, while deeper synovial regions appear relatively spared [123,124]. The loss of the capillary network is accompanied by increased erythrocyte extravasation, and both features correlate with hemoglobin A1c (HbA1c or glycosylated hemoglobin) levels. Hyperglycemia induces oxidative stress, inflammation, and mitochondrial dysfunction [125], leading to endothelial and pericyte apoptosis, loss of vascular stability, and increased capillary permeability [125,126].

Endothelial dysfunction in T2DM may arise both directly from chronic hyperglycemia and indirectly through T2DM-associated persistent low-grade systemic inflammation [127]. Pro-inflammatory cytokines elevated in T2DM, particularly IL-6 [46,127,128], disrupt endothelial intercellular adhesion and promote vascular leakage [127], while IL-6 concentrations observed in T2DM patients also inhibit capillary network formation [128].

Hypertension frequently coexists with T2DM, and it has also been independently associated with KOA [129]. Decreased peripheral blood flow due to hypertension has been proposed to cause impaired subchondral perfusion (subchondral ischemia), resulting in impaired nutrient and gas exchange with articular cartilage and eventual osteocyte death within subchondral bone [121]. In a large population-based analysis of 9514 individuals aged ≥50 years from the 5th Korean National Health and Nutrition Examination Survey (KNHANES), hypertension was significantly associated with radiographic KOA [129]. The prevalence of KOA was higher in hypertensive individuals compared with non-hypertensives, and this association remained statistically significant after full adjustment for confounding variables (fully adjusted odds ratio (OR) 1.26; 95% CI 1.08–1.48). Even after adjustment for age and sex, hypertensive subjects demonstrated increased odds of KOA (age- and sex-adjusted OR 1.48). They also found hyperglycemia to be strongly correlated with KOA [129].

The association between cardiovascular diseases and OA is also supported by the presence of a common transmembrane protein Z391Ig, which was found in both human carotid arterial plaque and osteoarthritic synovial lining macrophages [130].

These findings support the existence of shared inflammatory and vascular pathways between OA and cardiovascular disease. However, the precise contribution of vascular dysfunction to OA progression in patients with T2DM remains incompletely understood and requires further investigation.

Importantly, the strength of evidence varies among the mechanisms discussed above. Some pathways, such as chronic inflammation, AGE accumulation and microvascular dysfunction, have been directly investigated in diabetic OA models and human tissues. In contrast, mechanisms including ER stress, autophagy dysregulation, ferroptosis and stem cell dysfunction are often inferred from parallel observations in OA and T2DM or from high-glucose experimental systems. Because hyperglycemia represents only one component of the complex metabolic environment of T2DM, future studies using integrated diabetic OA models and human specimens are needed to establish causal relationships and determine the relative contribution of these pathways to disease progression.

## 4. Impact of T2DM on OA Prognosis

### 4.1. Arthroplasty Requirement as a Marker of Severe OA

Severe/symptomatic OA represents one of the most common indications for joint replacement surgery, particularly total hip and total knee arthroplasty [131]. Consistent with this, large population-based cohort studies indicate that T2DM is associated with an increased likelihood of undergoing total joint arthroplasty, supporting the use of arthroplasty requirement as a surrogate marker of severe OA [132]. In a study including approximately 450,000 individuals with T2DM and more than 3 million without T2DM, the rates of total joint arthroplasty were significantly higher in patients with T2DM, with ORs ranging from 1.2 for primary total knee arthroplasty to 3.4 for revision total hip arthroplasty. Notably, when stratified by age, the greatest relative risks were observed in younger diabetic patients (46–55 years) [132]. Similar findings were reported in another small-sample longitudinal study, in which T2DM was also positively associated with an increased risk of arthroplasty [38]. Together, those findings suggest that T2DM is associated with a more severe OA phenotype and an increased likelihood of progression to end-stage disease requiring surgical intervention [38,131,132].

### 4.2. Postoperative Outcomes in Patients with Diabetes

In addition to the increased need for arthroplasty, DM has also been associated with less favorable postoperative outcomes, since several studies have reported higher postoperative mortality rates among arthroplasty patients with T2DM compared with non-diabetic patients [133,134]. Furthermore, T2DM has been linked to an increased risk of postoperative complications, delayed recovery and poorer overall surgical outcomes, highlighting the clinical importance of metabolic comorbidities in patients undergoing joint replacement surgery [133,134].

Therefore, the impact of T2DM on OA extends beyond disease development and progression, potentially influencing both the need for surgical treatment and postoperative prognosis.

## 5. Genetic and Molecular Overlap Between T2DM and OA

Growing evidence indicates that T2DM and OA share overlapping genetic and molecular pathogenetic mechanisms that extend far beyond simple epidemiological comorbidity [18]. Clinical and experimental studies suggest that T2DM may contribute to OA development through alterations in signaling pathways that are essential for maintaining cartilage health homeostasis.

### 5.1. Shared Signaling Pathways

Among the pathways implicated in both T2DM and OA are the phosphatidylinositol kinase/phosphokinase B (PI3K/Akt)/mTOR) pathway which regulates cell survival, cellular metabolism, inflammation, autophagy and apoptosis [135] and the Hippo signaling pathway/Salvador–Warts–Hippo/Yes-associated protein (YAP)/transcriptional coactivator with a PDZ-binding domain (TAZ) (Hippo/YAP/TAZ) pathway, which plays a central role in chondrocyte mechanotransduction and the modulation of anabolic and catabolic activities of chondrocytes [136].

Additional support for a direct metabolic influence on cartilage originates from diabetic rat models, which demonstrate IGF-1 resistance in cartilage, suggesting that chronic hyperglycemia may shift chondrocyte behavior toward a degenerative phenotype [137].

### 5.2. AGE–RAGE Signaling as a Link Between T2DM and OA

Transcriptomic analyses have identified significant overlap in DEGs between T2DM and OA, particularly within AGE products/receptor for advanced glycation end-products (AGE–RAGE) and IL-17 signaling pathways [18].

In T2DM, chronic hyperglycemia promotes excessive accumulation of AGEs, which activate RAGE, and subsequently induce ER stress and inflammatory signaling [138]. AGEs additionally modify structural proteins through irreversible cross-linking reactions, increasing tissue stiffness and impairing the biomechanical properties of articular cartilage [139].

At the cellular level, AGE–RAGE interactions activate MAPK signaling pathways in chondrocytes, leading to increased production of MMPs and proinflammatory cytokines. Given that the protective role of PPARγ expression to maintain cartilage homeostasis is diminished, thereby the increased production of MMPs and proinflammatory cytokines is promoting cartilage breakdown [99,100,140,141]. Furthermore, hyperglycemia-induced AGE accumulation in synovial fibroblast-like cells can further intensify inflammation, indirectly accelerating cartilage degradation and OA progression [142].

### 5.3. IL-17 Signaling as a Shared Inflammatory Pathway

The IL-17 pathway represents an additional point of convergence between T2DM and OA [18]. Primarily produced by T-helper 17 cells [143], IL-17 contributes to host defense but is also implicated in the pathogenesis of chronic inflammatory and autoimmune diseases as well as several malignancies [144]. Patients with obesity and T2DM exhibit elevated circulating IL-17 levels [145,146]. Increased IL-17 activity promotes the expression of inflammatory mediators, including the receptor activator of nuclear factor kappa B ligand (RANKL) [147]. Increased IL-17 activity has also been implicated in several forms of inflammatory arthritis including OA and rheumatoid arthritis (RA) [148].

In OA, serum IL-17 concentrations correlate with cartilage defects, bone marrow lesions, and overall disease severity [149]. Mechanistically, IL-17 disrupts chondrocyte autophagy through activation of the PI3K/AKT/mTOR and Janus kinase/signal transducers and activators of transcription protein 3 (JAK/STAT3) signaling pathways, while simultaneously promoting premature cellular senescence [150]. In addition, IL-17 amplifies synovial inflammation by increasing the production of cytokines and chemokines such as IL-1, TNF-α, IL-6 and IL-8 [151].

### 5.4. Interaction Between AGE–RAGE and IL-17 Pathways

Emerging evidence suggests that AGE–RAGE and IL-17 signaling do not operate independently but rather participate in a broader proinflammatory network [18]. Transcriptomic analyses have identified Jun (JUN) and a C-X-C motif chemokine (CXCL)8 as shared key components of both AGE–RAGE and IL-17 pathways. These findings support the existence of a c-Jun(JUN)/CXCL8-centered inflammatory axis through which metabolic dysfunction may promote chronic inflammation and joint degeneration [18].

Collectively, shared alterations in gene expression, inflammatory signaling, cellular metabolism and tissue remodeling support the existence of a common molecular framework linking T2DM and OA. While some of these mechanisms have been directly demonstrated in diabetic OA models, others are inferred from parallel observations in OA and T2DM studies. Nevertheless, the available evidence supports the concept that metabolic abnormalities associated with T2DM may contribute to OA pathogenesis and progression through interconnected molecular and inflammatory pathways.

## 6. The Role of the PI3K/AKT/mTOR Pathway in OA Pathogenesis

The PI3K/AKT/mTOR signaling pathway regulates many cellular processes essential for homeostasis, including cell growth, proliferation, metabolism, survival, autophagy and apoptosis [152]. Dysregulation of this pathway contributes to the development of several chronic diseases, including obesity and T2DM, metabolic disorders in which impaired insulin signaling through the PI3K/AKT axis promotes insulin resistance, whereas alterations in mechanistic target of rapamycin complex 1 (mTORC1) signaling disrupt glucose and lipid metabolism [153,154,155]. Furthermore, chronic disturbances of mTORC1 signaling have been linked to impaired glucose homeostasis through effects on insulin signaling and hepatic gluconeogenesis [155,156,157,158].

The same pathway has also been implicated in OA pathogenesis [159]. Cartilage homeostasis depends on a tightly controlled balance between ECM synthesis and degradation [160]. Disruption of this balance is associated with increased expression of matrix-degrading enzymes, including MMPs and ADAMTSs, accompanied by reduced synthesis of COL2 and aggrecan [161,162,163]. These alterations contribute to the initiation and progression of OA [164]. Experimental studies have demonstrated that the PI3K/AKT/mTOR signaling pathway is an essential regulator of chondrocyte metabolism, autophagy, survival and overall cartilage homeostasis [112,113]. In OA models, aberrant activation of mTOR signaling suppresses autophagy and promotes cartilage degeneration, whereas genetic or pharmacological inhibition of mTOR has been shown to attenuate OA progression [165].

Importantly, evidence supporting the involvement of PI3/AKT/mTOR pathway in both T2DM and OA does not necessarily establish a direct mechanistic link between the two diseases. Much of the available evidence originates from independent studies of OA and T2DM or from hyperglycemia-based experimental models, which primarily mimic elevated glucose exposure rather than the complex metabolic environment of T2DM. Nevertheless, because the PI3/AKT/mTOR pathway regulates processes that are critical for both metabolic homeostasis and cartilage integrity, its dysregulation represents a biologically plausible mechanism through which T2DM may contribute to OA development and progression. Further studies using diabetic OA models and human tissues from patients with both T2DM and OA are required to establish the precise role of this pathway in the T2DM-OA interface.

## 7. Pain Perception, Neuropathy and Symptom Severity in T2DM and OA

OA patients have higher levels of pain perception even in the absence of T2DM, and this could be explained by mechanisms such as lower pressure pain thresholds and central sensitization [166]. OA patients with coexisting T2DM often report more severe joint pain [38,167]. The heightened pain sensitivity can be attributed to both local joint changes [38] and diabetic neuropathy [168,169]. In OA patients with T2DM, both synovitis and high IL-6 levels in the joint synovial fluid contribute to increased pain intensity [38,43,170,171].

T2DM can alter both peripheral and central nociceptive pathways. Diabetic peripheral neuropathy promotes pain amplification through nerve damage and aberrant sensory signaling, whereas central sensitization enhances the processing of nociceptive inputs within the central nervous system. Consequently, pain perception may be heightened independently of the extent of structural joint pathology [168,169,172]. Consequently, patients with both OA and T2DM may experience pain originating from a combination of joint pathology and neuropathic mechanisms.

Recent evidence suggests that structural remodeling of joint innervation, i.e., nociceptor sprouting or neo-innervation also contributes to altered pain signaling in OA [173]. Obeidat et al. [173] provided detailed explanation and evidence in this regard by using four murine models of KOA: destabilization of the medial meniscus (DMM), partial meniscectomy (PMX), non-invasive anterior cruciate ligament rupture (ACLR), and aging-related primary OA. Nociceptors were visualized using NaV1.8-tdTomato reporter mice and PGP9.5 immunostaining. The authors reported that the synovitis and the associated neo-innervation of the synovium by nociceptors peaked in early-stage OA. Similarly, in the subchondral bone, channels containing sprouting nociceptors appeared early and progressed with worsening joint damage. All four models showed increased nociceptive nerve signals within osteophytes. They are also of the view that anatomical neuroplasticity of nociceptors is an integral part of OA pathology [173].

Taken together, local joint inflammation, diabetic neuropathy, altered central pain processing and structural remodeling of joint innervation appear to interact in patients with T2DM and OA. These mechanisms likely contribute to increased pain sensitivity and may partly explain the more severe symptomatic phenotype observed in OA patients with coexisting T2DM [38,167,168,169,170,171,173].

## 8. Medication Effects: How Antidiabetic Drugs Influence OA

Antidiabetic medications may directly or indirectly influence the development and progression of OA. Beyond their metabolic effects, several antidiabetic agents modulate inflammatory signaling, autophagy, cartilage metabolism and tissue repair processes that are increasingly recognized as important in OA pathogenesis. Understanding these pharmacological interactions may facilitate the development of therapeutic strategies that simultaneously address metabolic dysfunction and joint degeneration.

### 8.1. Metformin

Metformin, a widely used first-line agent, exhibits anti-inflammatory and pro-autophagic effects, and multiple experimental and clinical studies suggest it may slow OA progression [174]. Its benefits appear to be mediated through activation of AMPK and downstream suppression of NF-κB signaling, as well as enhancement of chondrocyte autophagy [174,175,176,177]. Conversely, insulin therapy, particularly in hyperinsulinemic states, may accelerate OA progression due to its inhibitory effects on chondrocyte autophagy [53]. Understanding these pharmacologic interactions is essential for developing treatment strategies that address both metabolic control and joint health.

Metformin is also now emerging as a disease-modifying drug for treating OA in both human [178,179,180,181] and preclinical studies [135,182,183]. In a randomized clinical trial conducted by Pan and colleagues that involved KOA patients with obesity, metformin (2000 mg/day) resulted in an improvement in knee pain at 6 months in the group receiving metformin compared to the placebo group [178]. In another prospective cohort study utilizing KOA patient data from the Osteoarthritis Initiative, which involved KOA patients with obesity, metformin use was associated with a reduced rate of medial knee cartilage volume loss over a follow-up period of 4 years, as well as a trend toward a significant reduction in the risk of total knee replacement over 6 years [179]. Another retrospective study that compared the use of metformin combined with cyclooxygenase-2 (COX-2) inhibitors in patients with OA and comorbid T2DM to a control group not on metformin found the treated patients had a significantly lower rate of lower-limb total joint replacement, suggesting that metformin may slow disease progression or even have chondroprotective properties [180]. Baker et al. compared the incidence of OA and joint replacement surgeries in T2DM patients taking metformin or sulfonylureas [181]. They found out that T2DM patients in the metformin group had a significantly lower incidence of OA, but no significant differences were observed in joint replacement surgeries between the two groups [181].

In a preclinical experimental study involving a rat KOA model, metformin was shown to significantly ameliorate disease progression. This was histologically demonstrated by an increase in chondrocytes numbers, preservation of proteoglycans and reducing ECM degradation. Moreover, immunohistochemistry and Western blot analyses revealed reduced expression of catabolic markers (e.g., ADAMTS5) and a rise in anabolic cartilage-related markers (e.g., SRY-Box transcription factor 9 [SOX9] and COL2). Metformin also reversed OA-associated suppression of autophagy while downregulating phosphorylated PI3K, AKT and mTOR levels [135]. Moreover, metformin also showed promising results in additional mouse OA models by inhibiting ferroptosis, alleviating OA-associated microstructural changes in subchondral bone and reducing chondrocyte senescence [182,183].

### 8.2. Glucagon-like Peptide-1 Receptor Agonists

Incretins have also been explored recently as one of the treatment therapies for OA. Incretin-based therapies, such as glucagon-like peptide-1 receptor agonists (GLP-1 RAs), promote substantial weight loss and improve glycemic control in obesity and T2DM through mechanisms such as appetite suppression, delayed gastric emptying and glucose-dependent insulin secretion [184], as well as by reducing proatherogenic risk factors like hyperglycemia, hypertension and dyslipidemia [185,186]. They also improve endothelial function and vasodilation and reduce oxidative stress and inflammation [185]. GLP-1 RAs cause suppression of toll-like receptor (TLR)-induced production of proinflammatory cytokines (such as TNF-α), via α1-adrenergic, δ- and κ-opioid signaling pathways in the central nervous system. This further leads to a reduction in systemic inflammation [187].

In KOA, these therapies provide a novel strategy, primarily through weight loss, to alleviate pain and enhance physical activity, aligning with the American College of Rheumatology and Arthritis Foundation guidelines that prioritize ≥ 5% weight reduction for symptomatic improvement in overweight or obese individuals, with greater benefits at higher levels of weight reduction [188]. The STEP 9 trial recently provided evidence supporting this recommendation by evaluating semaglutide at a dose of 2.4 mg weekly in individuals with obesity and KOA [189]. Both the semaglutide and placebo groups received lifestyle interventions, including recommendations to increase physical activity and reduce caloric intake. Semaglutide led to significantly greater weight loss of 13.7%, compared to 3.2% in the placebo group, and improved the Western Ontario and McMaster Universities Osteoarthritis Index pain score [189]. However, in another randomized trial including subjects with KOA and a BMI ≥ 27 kg/m^2^, liraglutide at a dose of 3 mg daily did not significantly improve knee pain despite achieving a more significant weight loss than the placebo [190].

### 8.3. Corticosteroids and Metabolic Consequences

Intra-articular corticosteroid injections are commonly used for OA due to their anti-inflammatory and analgesic effects. However, systemic absorption may occur, resulting in endocrine complications. A published case report described a woman with OA who developed new-onset T2DM and clinical features of hypercortisolism following prolonged intra-articular corticosteroid therapy [191]. Further evaluation confirmed iatrogenic Cushing syndrome with secondary adrenal insufficiency attributable to repeated injections. Discontinuation of corticosteroids and glucocorticoid replacement therapy led to gradual recovery of adrenal and ovarian function [191]. This case highlights that intra-articular corticosteroids, despite local administration, may cause clinically significant hypothalamic–pituitary–adrenal axis suppression and metabolic disturbances.

Overall, antidiabetic therapies may substantially influence OA progression and symptom severity. Among currently available agents, metformin demonstrates the strongest evidence for potential disease-modifying effects through anti-inflammatory, pro-autophagic and chondroprotective mechanisms, while GLP-1 receptor agonists may provide additional benefits through weight reduction and systemic metabolic improvement. Future randomized clinical trials are needed to determine whether these therapies can be effectively integrated into personalized treatment strategies for patients with coexisting T2DM and OA.

## 9. Public Health Perspective

The coexistence of T2DM and OA may significantly amplify the economic burden on healthcare systems and society, increasing the already substantial costs. DM is a global economic burden, with a cost-of-illness analysis estimating its total worldwide cost at around USD 1.31 trillion, representing approximately 1.8% of global gross domestic product (GDP), with roughly one-third of this burden due to indirect costs such as lost productivity and premature mortality [192]. Meanwhile, OA imposes both high direct medical costs (e.g., pain management, joint replacement) and indirect costs (e.g., loss of work, reduced quality of life), with annual healthcare costs per OA patient ranging from €705 to over €19,700, and non-healthcare costs of up to €11,900; the social cost of OA has been estimated at 0.25–0.50% of a country’s GDP [193].

A recent cost-consequence analysis conducted in Saudi Arabia among 314,079 privately insured adults with obesity estimated that obesity-related conditions (such as T2DM, hypertension and OA) resulted in USD 1.245 billion in healthcare costs in 2023, with projections suggesting a doubling cost over 10 years [194]. The authors noted that a 15% weight loss could save USD 1.295 billion over the next 10 years, with T2DM being the biggest contributor to the projected savings (USD 430 million).

When T2DM and OA co-occur, healthcare utilization increases through greater pharmacologic needs, more frequent hospitalizations and a higher likelihood of surgery. Functional limitations from OA can hinder T2DM self-management, while T2DM complications reduce mobility—together with compounding productivity losses. Thus, the convergence of two high-cost chronic diseases signals a major and growing public health challenge, particularly in aging and increasingly obese populations.

## 10. Future Direction and Perspectives

### 10.1. Molecular Mechanisms and Translational Research

Despite growing evidence linking T2DM and OA, the underlying molecular mechanisms defining this interaction remain poorly ununderstood. Future research should focus on these mechanisms to characterize the shared susceptibility loci, such as *WSCD1* and *TMEM119*, to identify the specific effector genes, regulatory networks and epigenetic modifications involved in both diseases. Attention should be given to understanding how hyperglycemia influences gene expression, cellular metabolism and cartilage homeostasis.

Further investigation of emerging pathways, including AGE–RAGE signaling, IL-17-mediated inflammation, autophagy dysregulation and ferroptosis, may reveal novel therapeutic targets. In particular, the LGR6/β-catenin/NRF/GPX4 axis has emerged as a promising regulator of ferroptosis in diabetic OA and warrants further validation in both experimental and clinical settings. An improved understanding of the interactions between inflammatory, metabolic and catabolic pathways may facilitate the identification of biomarkers capable of predicting OA progression in patients with T2DM.

### 10.2. Clinical Translation and Therapeutic Trials

The overlap between metabolic and musculoskeletal pathways offers opportunities for drug repurpose and the development of disease-modifying therapies. Antidiabetic agents such as metformin and GLP receptor agonists, including semaglutide, have shown promising anti-inflammatory and chondroprotective properties and should be further evaluated in well-designed, randomized, controlled trials.

Future clinical studies should focus not only on symptom improvement but also on structural disease modification. Long-term trials incorporating advanced imaging modalities, particularly MRI, could assess cartilage integrity, synovial inflammation, subchondral bone changes and disease progression over time. In parallel, therapeutic approaches targeting specific molecular pathways, such as IL-17 inhibition, autophagy enhancement through mTOR modulation or ferroptosis suppression, may represent innovative treatment strategies for patients suffering from T2DM and OA.

Given the increasing prevalence of both T2DM and OA, integrated healthcare approaches are required to address this growing multimorbidity. Future efforts should focus on implementing multidisciplinary care models involving endocrinologists, rheumatologists, orthopedic specialists, physiotherapists, dietitians and primary care physicians. Such models include coordinated screening for OA symptoms in patients with T2DM, routine assessment of metabolic health in OA patients, individualized exercise programs, nutritional counseling and weight-management systems.

Rather than focusing on numerous emerging technologies, future digital health applications should concentrate on practical tools that support long-term disease management, such as activity monitoring and remote tracking of glycemic control and physical function. These approaches may facilitate patient engagement, improve adherence to lifestyle interventions and enable earlier identification of individuals at high risk of disease progression.

In addition, health-economic studies are needed to evaluate the cost-effectiveness of preventive strategies, including weight-management programs and early identification of metabolic OA phenotypes. Ultimately, combining mechanistic research with targeted clinical interventions and coordinated multidisciplinary care may improve outcomes and help reduce the growing global burden of both T2DM and OA.

## 11. Conclusions

T2DM and OA are highly prevalent chronic disorders that frequently coexist and share important epidemiological, clinical and biological links. Growing evidence indicates that T2DM contributes to OA development and progression beyond the effects of aging and obesity alone, resulting in increased disease severity, pain burden and risk of joint replacement.

Recognizing OA and T2DM as interconnected disorders has important implications for patient care. Early identification of metabolic risk factors, optimization of glycemic control, weight management and interdisciplinary management may improve outcomes in patients affected by both conditions. Furthermore, emerging evidence supporting the chondroprotective effects of antidiabetic therapies highlights the opportunity for therapeutic repurposing.

Future research should focus on validating shared molecular pathways, identifying biomarkers of metabolic OA and evaluating targeted interventions in well-designed clinical trials. A better understanding of the interactions between metabolic dysfunction and joint disease may facilitate the development of personalized therapeutic strategies and integrated care models aimed at reducing the growing health and socioeconomic burden associated with both T2DM and OA.

## Figures and Tables

**Figure 1:**
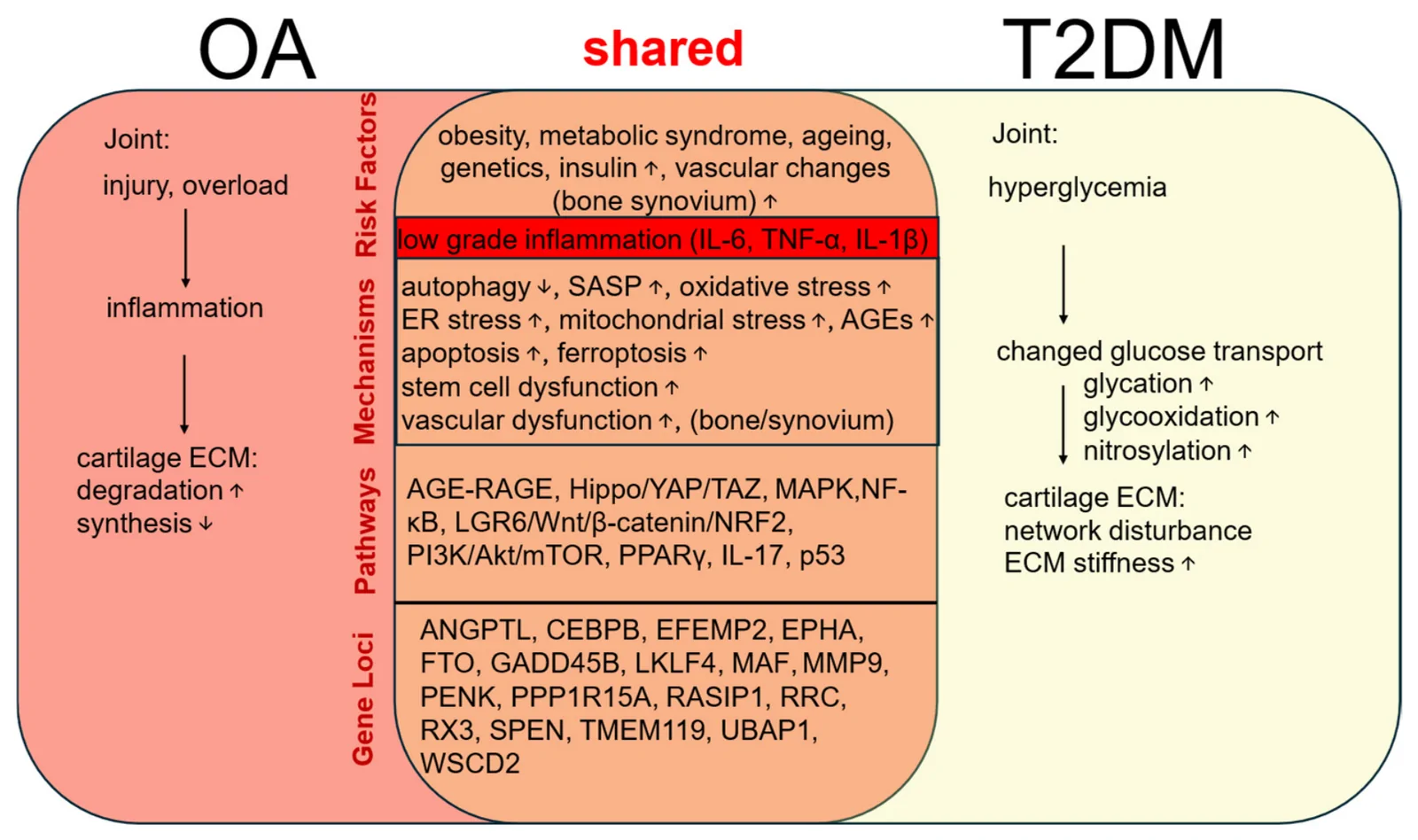
Shared risk factors, mechanisms, pathways and gene loci between OA and T2DM. AGEs: advanced glycation end-products; AGE–RAGE: advanced glycation end-products–receptor for advanced glycation end-products; ANGPTL: angiopoietin-like; CEBPB: CCAAT/enhancer-binding protein beta; ECM: extracellular matrix; EFEMP2: EGF-like fibulin extracellular matrix protein; EPHA: ephrin type-A receptor; FTO: fat mass and obesity-associated gene; GADD45B: growth arrest and DNA damage-inducible beta; Hippo/YAP/TAZ: Hippo signaling pathway/Salvador-Warts-Hippo/Yes-associated protein/transcriptional coactivator with a PDZ-binding domain; IL-6: interleukin 6; IL-1β: interleukin 1β; IL-17: interleukin 17; IRX3: Iroquois homeobox 3; KLF4: Krüppel-like factor; LGRR6/Wnt/β-catenin/NRF2: leucine-rich repeat-containing/Wingless-related integration proteins/β-catenin/nuclear factor erythroid 2-related factor 2; LRRC: leucine-rich repeat-containing; MAF: v-maf avian musculoaponeurotic fibrosarcoma oncogene homolog; MAPK: mitogen-activated protein kinase; MMP-9: matrix metalloproteinase 9; NF-κB: nuclear factor “kappa-light-chain-enhancer” of activated B-cells; PENK: proenkephalin; PI3K/Akt/mTOR: phosphatidylinositol kinase/protein kinase B/mechanistic target of rapamycin; PPARγ: peroxisome proliferator-activated receptor gamma; PPP1R15A: protein phosphatase 1 regulatory subunit 15A; p53: protein 53; RASIP1: RAS interaction protein; SASP: senescence-associated secretory phenotype; SPEN: solid pseudopapillary epithelial neoplasm; TMEM119: transmembrane protein 119; TNF-α: tumor necrosis factor-α; UBAP1: ubiquitin-associated protein 1 and WSCD2: WSC domain-containing 2; ↑: increase; ↓: decrease.

**Figure 2:**
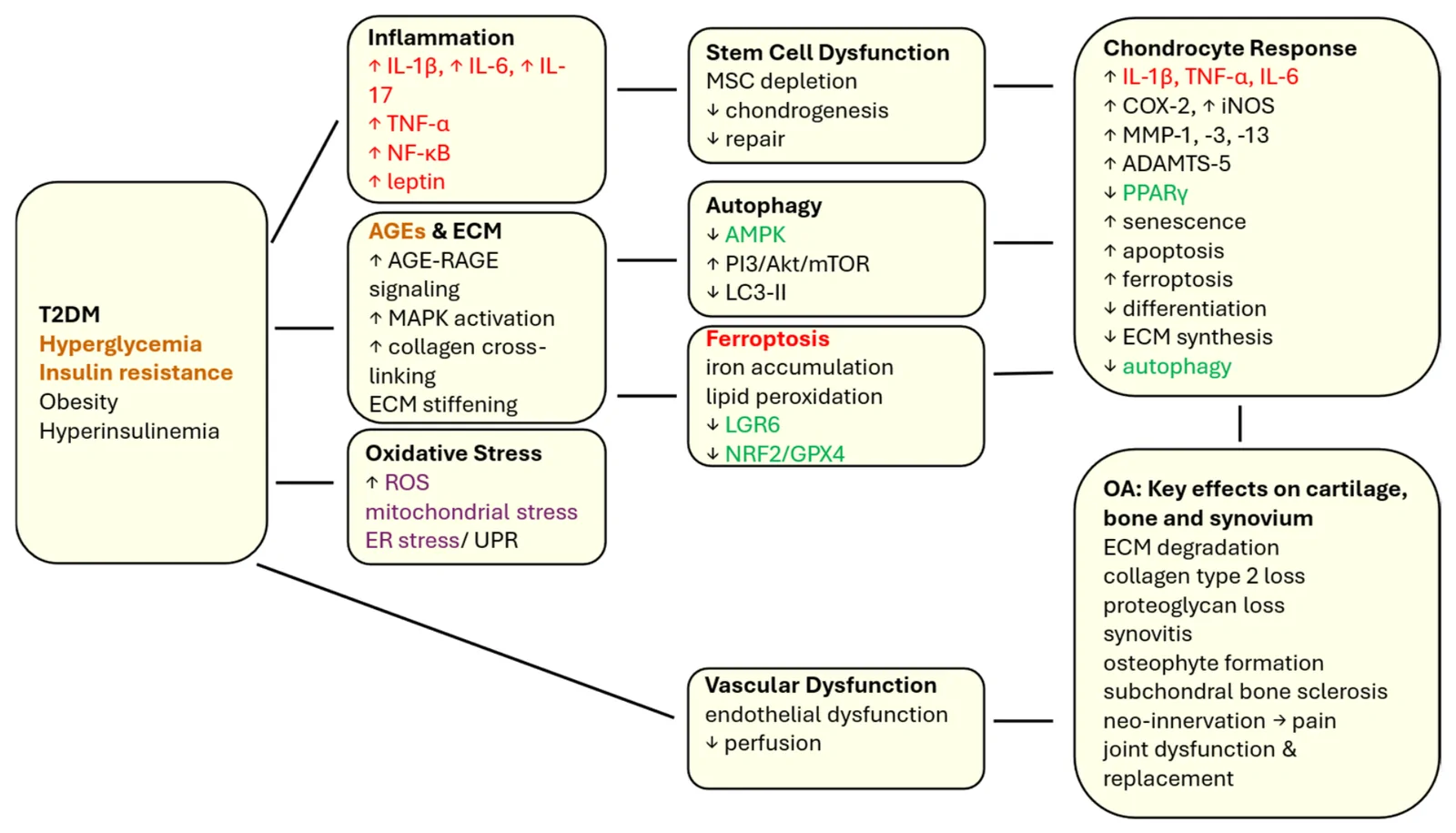
Shared molecular and cellular mechanisms linking T2DM and OA. T2DM accelerates OA progression via chronic inflammation, oxidative stress, AGE accumulation, autophagy suppression, stem-cell dysfunction, ferroptosis and microvascular impairment that ultimately disrupts cartilage homeostasis and promotes joint degeneration. ADAMTS: a disintegrin and metalloprotease with thrombospondin motifs; AGE: advanced glycation end-products; AMPK: adenosine monophosphate protein kinase; COX-2: cyclooxygenase-2; ECM: extracellular matrix; ER: endoplasmic reticulum; IL: interleukin; iNOS: inducible nitric oxide; LC3-II: microtubule-associated protein 1 light chain 3; LGR6: leucine-rich repeat-containing G-protein-coupled receptor 6; MAPK: mitogen-activated protein kinase; MMP: matrix metalloproteinase; MSC: mesenchymal stem cells; NF-κB: nuclear factor kappa-light-chain-enhancer of activated B-cells; NRF2/GPX4: nuclear factor erythroid 2-related factor 2/glutathione peroxidase 4; OA: osteoarthritis; PI3/Akt/mTOR: phosphatidylinositol kinase/protein kinase B/mechanistic target of rapamycin; PPARγ: peroxisome proliferator-activated receptor gamma; RAGE: receptor for advanced glycation end-products; ROS: reactive oxygen species; T2DM type 2 diabetes mellitus; TNF-α: tumor necrosis factor-α; UPR: unfolded protein response;. Color coding: red: damaging processes; orange: metabolic stress; violet: cell stress; green: protective pathways; ↑: increase; ↓: decrease.

## Data Availability

No new data were created or analyzed in this study. Data sharing is not applicable to this article.

## Abbreviations

The following abbreviations are used in this manuscript:

ACLR	anterior cruciate ligament rupture
ADAMTSs	a disintegrin and metalloprotease with thrombospondin motifs
ADSCs	adipose derived stem cells
AGEs	advanced glycation end-products
AGE–RAGE	AGE products/receptor for advanced glycation end-products
Akt	protein kinase B
AMPK	adenosine monophosphate protein kinase
ANGPTL	angiopoietin-like
ATF6	activating transcription factor 6
BMI	body mass index
BMP2	bone morphogenetic protein 2
BRI	Body Roundness Index
CDC	Centers of Disease Control and Prevention
CEBPB	CCAAT/enhancer-binding protein beta
COL2	collagen type II
COX-2	cyclooxygenase-2
CSPCs	cartilage stem/progenitor cells
CXCL	C-X-C motif chemokine
DEGs	differentially expressed genes
DM	diabetes mellitus
DMM	destabilization of the medial meniscus
ECM	extracellular matrix
EFEMP	EGF-like fibulin extracellular matrix protein
EPHA	ephrin type-A receptor
ER	endoplasmic reticulum
FFAs	free fatty acids
FTO	fat mass and obesity-associated gene
GADD45B	growth arrest and DNA damage-inducible beta
GDP	global gross domestic product
GLP-1 RAs	glucagon-like peptide-1 receptor agonists
GLUT1	glucose transporter 1
GPX4	glutathione peroxidase 4
HbA1c	hemoglobin A1c or glycosylated hemoglobin
Hippo	Hippo signaling pathway/Salvador–Warts–Hippo
HOA	hip OA
HRQoL	health-related quality of life
IFP	infrapatellar fat pad
IGF	insulin-like growth factor
IL	interleukin
iNOS	inducible nitric oxide
IRE1α	inositol-requiring enzyme 1 alpha
IRX3	Iroquois Homeobox 3
JAK/STAT3	Januskinase/Signal Transducers and Activators of Transcription protein 3
JUN	c-Jun
KLF	Krüppel-like factor
KNHANES	Korean National Health and Nutrition Examination Survey
KOA	knee osteoarthritis
LC3 II	microtubule-associated protein 1 light chain 3
LGR6	leucine-rich repeat-containing G-protein coupled receptor 6
LPO	lipid peroxidation
LRRC	leucine rich repeat containing
MAF	v-maf avian musculoaponeurotic fibrosarcoma oncogene homolog
MAPK	mitogen-activated protein kinase
METS-VF	visceral fat metabolism score
MMP	matrix metalloproteinase
MR	Mendelian randomization
MRI	magnetic resonance imaging
MSCs	mesenchymal stem cells
mTOR	mechanistic target of rapamycin
mTORC1	mechanistic target of rapamycin complex 1
MUFAs	monounsaturated fatty acids
NF-κB	nuclear Factor kappa-light-chain-enhancer of activated B-cells
NHANES	National Center for Health Statistics of the Centers for Disease Control and Prevention
NO	nitric oxide
NOS2	nitric oxide synthase-2
NRF2	nuclear factor erythroid 2-related factor 2
OA	osteoarthritis
OR	odds ratio
p53	protein 53
PD	prediabetes
PENK	proenkephalin
PERK	protein kinase RNA-like ER kinase
PGs	prostaglandins
PGE2	prostaglandin E2
PI3K/Akt	phosphatidylinositol kinase/phosphokinase B
PMX	partial meniscectomy
PPARγ	peroxisome proliferator-activated receptor gamma
PPI	protein–protein interaction
PPP1R	protein phosphatase 1 regulatory subunit
p-rpS6	phosphorylated ribosomal protein S6
PTGS2	prostaglandin-endoperoxide synthase 2
PUFAs	polyunsaturated fatty acids
RA	rheumatoid arthritis
RANKL	receptor activator of nuclear factor kappa B ligand
RASIP	RAS interaction protein
ROS	reactive oxygen species
rpS6	ribosomal protein S6
SFAs	saturated fatty acids
SOX9	SRY-Box transcription factor 9
SPEN	solid pseudopapillary epithelial neoplasm
T2DM	type 2 diabetes mellitus
T3	triiodothyronine
T4	thyroxin
TAZ	transcriptional coactivator with a PDZ-binding domain
TLR	toll-like receptor
TMEM119	transmembrane protein 119
TNF-α	tumor necrosis factor alpha
UBAP	ubiquitin-associated protein
UPR	unfolded protein response
Wnt	wingless-related integration proteins
WSCD2	wall integrity and stress component domain containing 2
YAP	Yes associated protein
